# CBCT-Based Orthodontic Classification Using Commercial AI: Completeness and Accuracy in Independent Validation

**DOI:** 10.3390/jcm15041637

**Published:** 2026-02-21

**Authors:** Natalia Kazimierczak, Nora Sultani, Szymon Krzykowski, Zbigniew Serafin, Wojciech Kazimierczak

**Affiliations:** 1 Kazimierczak Clinic, Dworcowa 13/u6a, 85-009 Bydgoszcz, Poland; 2 Faculty of Medicine, Collegium Medicum, Nicolaus Copernicus University in Torun, Jagiellońska 13-15, 85-067 Bydgoszcz, Poland; 3 Faculty of Medicine, Bydgoszcz University of Science and Technology, Kaliskiego 7, 85-796 Bydgoszcz, Poland

**Keywords:** artificial intelligence, CBCT, orthodontics, cephalometry, diagnostic accuracy, machine learning

## Abstract

**Background/Objectives**: Artificial intelligence (AI) tools for orthodontic diagnosis are increasingly used in clinical practice; however, there is limited evidence regarding their performance in CBCT-based assessments. In this study, we evaluated the diagnostic reliability of the Diagnocat platform for categorical orthodontic diagnoses obtained from CBCT examinations. **Methods**: Fifty-nine patients who underwent large-field CBCT (13 × 16 cm) and lateral cephalograms within 30 days were included, and CBCT scans were processed using Diagnocat (v1.0). The platform’s categorical outputs—sagittal skeletal class, vertical facial pattern, overbite category, and Dental Angle class—were compared with manual cephalometric analyses performed by an experienced orthodontist (reference standard). Standard thresholds were used to convert reference continuous measurements into categorical variables. Missing or ‘N/A’ index test outputs were treated as diagnostic failures in accordance with STARD recommendations. Agreement was assessed via Cohen’s kappa (κ), and the sensitivity, specificity, PPV, and NPV were calculated for angle classification. **Results**: The AI platform generated skeletal and vertical classifications in only 3/59 (5%) and 1/59 (1.7%) patients, respectively. Agreement was fair (κ = 0.324) for overbite categorization, and the Dental Angle class was provided for 34/59 (57.6%) patients. When “N/A” results were treated as diagnostic failures, the overall system usability was <10% for skeletal parameters. **Conclusions**: The platform demonstrated insufficient diagnostic reliability and failed to generate outputs for most patients. While the specificities for generated diagnoses were acceptable, the low data completeness rate renders the tool currently unsuitable for independent clinical decision-making.

## 1. Introduction

In 1930, Broadbent and Hofrath developed a new standardized method to obtain lateral cephalometric radiographs, and since then, cephalometric analysis (CA) has become a milestone in orthodontics [1]. CA enables precise assessment of dentofacial morphology and provides a reliable diagnosis of malocclusion and its anatomical basis, which supports precise diagnostics and treatment planning. In addition, CA makes it possible to analyze craniofacial growth through morphometric methods and inspect treatment progress [2]. In orthognathic surgery, CA plays a dual role with respect to its use in orthodontics and enables precise surgical treatment to be planned and treatment results to be monitored [3].

Conventional cephalometric analysis (CA) relies on two-dimensional (2D) lateral cephalograms of the craniofacial complex to obtain precise linear and angular measurements between skeletal and soft-tissue landmarks. However, conventional human-reading CA has several drawbacks, such as dependence on the examiner’s skills, variability in landmark identification, and a time-consuming workflow [4]. In recent years, artificial intelligence (AI) has attracted significant attention in medicine, particularly in diagnostic imaging. Because orthodontic treatment planning requires radiological data, automated cephalometric analysis has become one of the fastest-evolving applications of AI [5]. Compared with human-reader analysis, AI-driven CA reduces operator dependency and reduces inconsistencies in landmark detection [6,7].

The first attempts to introduce CT and CBCT into CA appeared in the early 2000s, but their time consumption and low efficiency limited their widespread use [8]. Advances in AI, including automated three-dimensional skull assessments, have gained popularity in tomography-based cephalometry. Multiple investigations have demonstrated that AI-based landmark detection and cephalometric measurements achieve high accuracy and efficiency compared with traditional human analyses [9,10,11,12]. Moreover, studies have shown that both automated 2D and 3D analyses can be completed within a minute, highlighting their time efficiency [13].

The growing number of companies developing AI-driven tools for healthcare highlights its presence in the commercial market, and AI applications are now available not only for research but also as commercial web-based platforms. In orthodontics, software such as Invivo7 Anatomage (Version 7.2.3, Osteoid, Inc., Santa Clara, CA, USA) and CephX (ORCA Dental AI, Las Vegas, NV, USA) provide CBCT-based cephalometric analyses. Despite their increasing commercial presence, most of these AI systems are proprietary and lack transparency regarding their methodologies. Therefore, independent validation studies are very important to confirm the clinical accuracy and reliability of these methods.

Most research focuses on developing and benchmarking deep learning models (commonly convolutional architectures) under research conditions, whereas clinicians increasingly rely on proprietary commercial systems whose categorical diagnostic logic and confidence thresholds are not transparent. Therefore, clinicians are increasingly presented with “black-box” commercial solutions that integrate directly into practice management software. Unlike research models, these commercial tools are rarely subjected to independent external validation regarding their categorical diagnostic logic. Therefore, the aim of this study was not to compare different machine learning architectures but to perform a clinical audit of a specific, widely available commercial platform (Diagnocat) to determine its reliability and data completeness in a real-world orthodontic workflow.

In this study, we aimed to evaluate the reliability of Diagnocat, a web-based platform, for AI-automated orthodontic diagnosis via cone-beam computed tomography (CBCT) images. The AI-generated diagnoses were compared with the reference standard, the cephalometric analysis performer, on lateral cephalograms by experienced clinicians.

## 2. Materials and Methods

### 2.1. Study Design and Ethics

This was a retrospective study on diagnostic accuracy with a cross-sectional design, evaluating the categorical orthodontic outputs of a commercial AI system generated after CBCT upload against a prespecified reference standard. It was conducted in accordance with the Declaration of Helsinki and approved by the bioethics committee of Collegium Medicum, Nicolaus Copernicus University (IRB approval number: KB 274/2025; date of approval: 23 April 2025). All imaging data were de-identified prior to analysis, and the need for informed consent was waived according to the ethics decision and retrospective nature of the study.

### 2.2. Setting and Participants

A total of 150 consecutive patients who were referred for orthodontic evaluation at a private dental practice between 01.2024 and 04.2025 were screened. The inclusion criteria were as follows:

(1) Availability of a diagnostic CBCT scan with a field of view (FOV) of 13 × 16 cm and a lateral cephalogram acquired within ≤30 days;
(2) Image quality sufficient for analysis.

The exclusion criteria were craniofacial syndromes or cleft lip/palate and severe motion or metal artifacts compromising interpretation.

**Figure 1:**
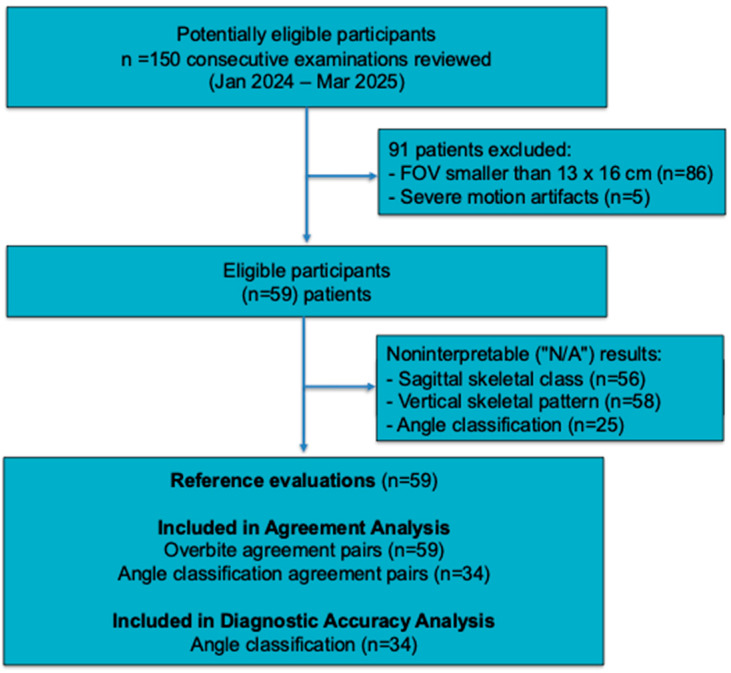
STARD flow diagram.

After applying the eligibility criteria, 86 patients whose FOV was lower than 13 × 16 cm were excluded, with an additional 5 excluded because of severe motion artifacts. Finally, a total of 59 patients with both lateral cephalograms and CBCT images were included in the analysis. A STARD flow diagram summarizing patient selection, exclusions, and analysis sets is provided in Figure 1.

### 2.3. Image Acquisition

CBCT examinations were performed using a Hyperion X9 PRO unit (MyRay, Imola, Italy) with 90 kVp, 36 mAs, and a 13 × 16 cm FOV and reconstructed with a 0.3 mm slice thickness. Patients were positioned according to the manufacturer’s recommendations, with maximum intercuspation and a natural head position. The primary indication for large-field-of-view (FOV) CBCT imaging was orthognathic treatment planning.

Lateral cephalometric radiographs were also acquired with a Hyperion X9 PRO unit following standard positioning (Frankfort horizontal parallel to the floor, teeth in maximum intercuspation, lips at rest). The exposure parameters were set at 82 kV, 12 mA, 5.5 exposition time, and 15 mGy × cm^2DAP^, and all DICOM files were anonymized, coded, and exported for analysis. No adverse events occurred during image acquisition or data processing.

### 2.4. AI Evaluation

De-identified CBCT datasets were uploaded to Diagnocat (version 1.0., Diagnocat Ltd., San Francisco, CA, USA), which utilizes a proprietary Convolutional Neural Network (CNN) architecture. As a commercial “Software as a Service” (SaaS), the specific training datasets, layer depth, and internal confidence thresholds are not public. This “black box” nature is representative of the current commercial landscape.

The platform’s automated orthodontic module generates standardized outputs, which are exported as structured reports. The following variables were extracted for analysis (as present in the dataset):

Dental Angle class (I/II/III);

Overbite [and/or overjet if reported];

Skeletal class/relationships [if present].

**Figure 2:**
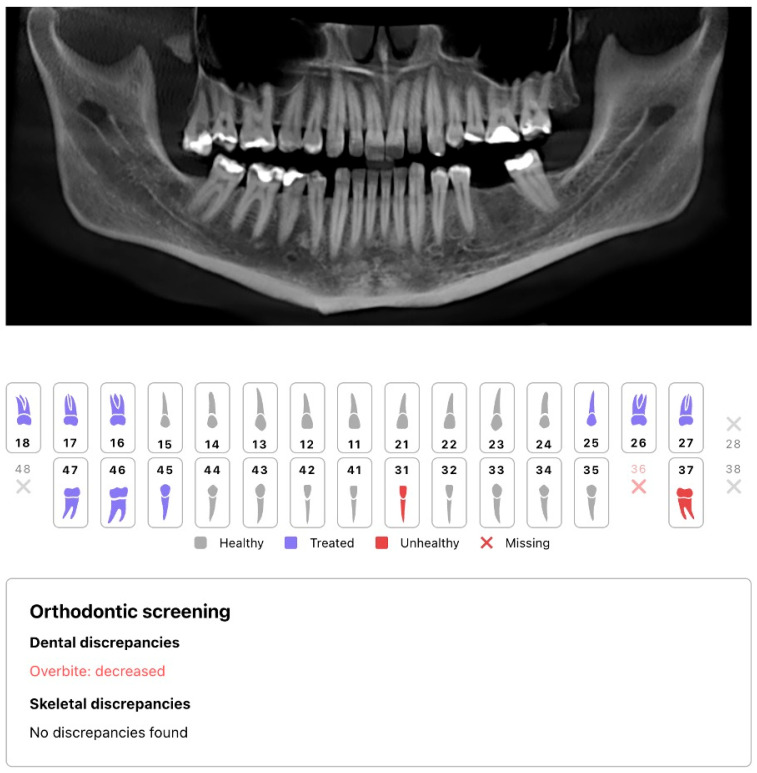
Sample orthodontic report of a 48-year-old female.

Index-test outputs were locked prior to reference assessments to maintain blinding. A sample AI-automated orthodontic report is presented in Figure 2.

Importantly, the Diagnocat v1.0 orthodontic report provided categorical outputs (and, in many cases, “N/A/No diagnosis”) but did not provide exportable 3D landmark coordinates, intermediate measurements (e.g., SNA/SNB/ANB), or documentation describing whether each categorical output was computed from the 3D CBCT volume, CBCT-derived 2D reconstructions (e.g., panoramic-like projections), or a hybrid workflow. Consequently, the present study was designed to validate the clinical output layer—data completeness and agreement of categorical diagnoses—rather than the upstream landmark localization performance. The inability to interrogate the internal computational pathway is an inherent limitation of proprietary “black-box” systems.

### 2.5. Reference Standard

One experienced board-certified orthodontist with over 10 years of experience in cephalometric analysis evaluated the cephalometric images. Manual 2D cephalometric diagnosis was selected as the reference standard because it remains the accepted clinical benchmark for sagittal, vertical, and dental classification, with extensive validation and widespread orthodontic adoption. The manual, digital cephalometric analysis was conducted with Ortobajt (ver. 9.2.5, Ortobajt, Wrocław, Poland).

Skeletal AP: SNA, SNB, ANB; Wits appraisal;

Vertical pattern: SN-MP (or SN-GoGn), FMA, Y-axis;

Dentoalveolar: U1-SN, L1-MP, interincisal angle;

Overjet/overbite (mm);

Soft-tissue (optional): N-A-Pog (facial convexity), E-line distances.

To assess intra-observer reliability, 12 randomly selected cases (20% of the sample) were re-digitized by the same examiner after a two-week washout period. The intraclass correlation coefficient (ICC) was calculated for continuous variables, and Kappa was calculated for categorical variables.

### 2.6. Data Processing and Categorization

Data from the reference standard, presented as continuous variables (e.g., angular measurements in degrees, linear in millimeters), were converted into categorical variables to allow for direct comparison with the qualitative results generated by the Diagnocat software v1.0. The categorization process was based on widely accepted clinical norms from the scientific literature [14].

Sagittal skeletal relationship: For the primary analysis, sagittal skeletal class was categorized from the ANB angle using prespecified thresholds (Class I: 0–4°; Class II: >4°; Class III: <0°). Because ANB may be influenced by cranial-base geometry and nasion position, we additionally prespecified a sensitivity analysis using the Wits appraisal to test the robustness of the reference categorization. In this sensitivity analysis, cases were categorized as Class I when Wits was between −2 and +2 mm, Class II when Wits was >+2 mm, and Class III when Wits was <−2 mm [15]. The primary results are reported using ANB-based categories.

**Vertical Facial Pattern:** The AI’s categorical assessment (facial pattern), where the descriptor “retro inclination” was interpreted as a hyperdivergent pattern, was compared with the categorized FMA angle (Frankfort–mandibular plane angle). The following thresholds were applied:

**Hypodivergent:** FMA < 22°;

**Normodivergent:** FMA ≥ 22° and ≤28°;

**Hyperdivergent:** FMA > 28°.

**Overbite:** The AI’s categorical assessment (Overbite) was compared with the categorized Overbite measurement (in mm) from the reference dataset. The following thresholds were applied:

**Normal:** Overbite ≥ 2 and ≤4 mm;

**Increased (deep):** Overbite > 4 mm;

**Decreased (open):** Overbite < 2 mm.

**Dental Angle Class:** The AI’s categorical assessment (Dental Angle class) was compared with the results of the reader assessment [16]:

Class I: The maxillary first molar mesiobuccal cusp occludes the mandibular first molar buccal groove, and the maxillary canine cusp is in the embrasure between the mandibular canine and first premolar.

Class II: The maxillary first molar is positioned anterior to Class I, and the canine cusp lies anterior to Class I.

Class III: The maxillary first molar is positioned posterior to Class I, and the canine cusp is posterior to Class I.

The reader was fully blinded to all index test outputs throughout data collection and re-evaluation. Likewise, Diagnocat index test outputs were generated automatically before human review, ensuring mutual blinding between assessors.

### 2.7. Handling of Missing Outputs

A critical distinction was made between diagnostic accuracy and data completeness:

**Completeness Rate:** Defined as the percentage of cases where the AI generated a definitive classification (excluding “N/A” or “No Diagnosis”).

**Agreement Analysis:** Performed only on the subset of cases where both the AI and Human Reference provided a classification.

**Accuracy:** For the angle classification, “N/A” outputs were penalized as “incorrect” to reflect the clinical utility of the system.

In accordance with STARD recommendations, noninterpretable (‘N/A’) index test outputs were treated as diagnostic failures for calculations of diagnostic accuracy. Since the platform did not provide any rationale for outputs labeled as “N/A” (i.e., no diagnostic classification returned), we prespecified that “N/A” would be treated as a test failure and therefore as an incorrect result for diagnostic accuracy calculations. Accordingly, diagnostic accuracy metrics were computed on the full cohort (*n* = 59) to reflect real-world clinical utility, where the inability to generate a diagnosis constitutes a non-actionable output.

### 2.8. Sample Size

This was a retrospective validation study, and the sample size was constrained by the number of eligible paired CBCT–cephalogram examinations available within the study period. Nevertheless, we performed an a priori-style precision-based sample size calculation for the primary reliability endpoint (Cohen’s κ), which is the recommended planning framework for agreement studies focused on interval estimation.

Using the large-sample standard error of κ, the approximate 95% CI half-width is

$$w\approx 1.96\sqrt{\frac{P_{o}(1-P_{o})}{n(1-P_{e}{)}^{2}}},$$

where $P_{o}$ is the expected observed agreement and $P_{e}$ is the expected chance agreement derived from the marginal category proportions.

For overbite (three categories; reference prevalence 0.254/0.424/0.322, yielding $P_{e}=0.348$), assuming κ ≈ 0.35, we obtain $P_{o}=0.576$ and a required sample size of *n* ≈ 56 to achieve w=0.20.

For Dental Angle class (including “N/A” as a usability category; estimated $P_{e}\approx 0.24$), assuming κ ≈ 0.30, *n* ≈ 42 achieves w=0.20.

Therefore, the final cohort size (*n* = 59) provides adequate precision (approximately ±0.17–0.19) for κ estimation in this exploratory independent validation.

### 2.9. Statistical Analysis

Statistical analysis was performed to assess the agreement and correlation between the two diagnostic methods.

**Agreement assessment:** To evaluate the degree of agreement between the categorized results of both methods for sagittal relationships, vertical patterns, and overbite, **Cohen’s kappa (κ)** statistic was used. Kappa values were interpreted according to the Landis and Koch scale, where values of 0.00–0.20 indicate slight agreement, 0.21–0.40 indicate fair agreement, 0.41–0.60 indicates moderate agreement, 0.61–0.80 indicates substantial agreement, and 0.81–1.00 indicates almost perfect agreement.

**Diagnostic Accuracy Assessment:** For the binary classification of the presence of Class II and Class III malocclusions, 2 × 2 contingency tables were constructed, and the sensitivity, specificity, positive predictive value (PPV), and negative predictive value (NPV) were calculated.

All accuracy estimates were accompanied by 95% confidence intervals generated using Wilson or exact methods where appropriate. The concordance of the quantitative measures between the raters was assessed with an intraclass correlation coefficient of type 3 (according to Shrout and Fleiss). We calculated 95% confidence intervals for Cohen’s κ using nonparametric bootstrap resampling. All the analyses were conducted in R software, version 4.5.1.

## 3. Results

### 3.1. Study Population

A total of 59 patients were included in this study, with a mean age of 27.2 (SD 11.1) years, median age of 25.0 years, and interquartile range of 18.0–35.0 years (range, 10.0–58.0 years). The sex distribution was 39 (66.1%) females and 20 (33.828%) males. No orthodontic treatment or dental procedures occurred between the CBCT and cephalometric acquisitions, as confirmed by clinical records.

### 3.2. Data Completeness

The primary finding was the system’s low data completeness rate. The skeletal class was generated in 3/59 cases (5.1%; 95% CI 1.7–13.9) and vertical pattern in 1/59 cases (1.7%; 95% CI 0.3–9.0), and the angle class was generated in 34/59 cases (57.6%; 95% CI 44.9–69.4). Owing to the extremely small number of available AI outputs for skeletal and vertical assessments, agreement statistics for these domains were not estimable with meaningful precision.

### 3.3. Agreement in Sagittal Skeletal Relationship Assessment

In the analyzed sample of 59 patients, manual cephalometric analysis identified 21 cases (35.6%) as Class I, 29 cases (49.2%) as Class II, and 9 cases (15.2%) as Class III. The Diagnocat software identified three cases that had corresponding skeletal descriptors. The comparison of classifications is presented in Table 1.

Owing to the negligible number of cases (3 out of 59) with a skeletal diagnosis by the Diagnocat software, the calculation of a reliable Kappa statistic and diagnostic accuracy parameters was not possible. In the three cases where the AI provided a diagnosis, it was 100% consistent with the reference method. However, in 56 out of 59 cases (95%), the software did not provide a skeletal classification, despite the presence of malocclusions in the reference data (29 Class II cases and 8 Class III cases).

### 3.4. Agreement in Vertical Facial Pattern Assessment

The reference analysis revealed that 15 patients (25.4%) had a hypodivergent pattern, 19 (32.2%) had a normodivergent pattern, and 25 (42.3%) had a hyperdivergent pattern. The Diagnocat software identified only one case (C051) as “retro inclination,” which was interpreted as a hyperdivergent pattern. The reference analysis for this patient confirmed this diagnosis (FMA = 31.05°). Owing to the lack of classification in the remaining 58 cases (98.3%), a statistical evaluation of agreement was not possible.

### 3.5. Agreement in Overbite Assessment

The comparison of overbite categorization revealed fair agreement between the Diagnocat software and the reference standard (κ = 0.324). The detailed results are presented in Table 2.

The software most frequently classified the overbite as “decreased” (35 cases), which was consistent with the reference in only 11 cases. The AI incorrectly classified 14 cases with a normal overbite and 11 with an increased overbite as decreased. The agreement for the “increased” category was higher—5 out of 10 cases were correctly identified.

### 3.6. Dental Angle Class Validation

The Diagnocat software assigned the Angle’s malocclusion class to 34 of the 59 patients (Class I: 19; Class II: 8; Class III: 7), but did not provide results for the remaining patients (recorded as N/A). The reasoning behind not providing the results of the Dental Angle calculation was not provided by the algorithm. However, the human reader did not assess 15 cases: 10 cases lacked first permanent molars; 4 cases presented mixed dentition with at least one first primary molar; and 1 case exhibited no molar intercuspation due to malocclusion. All the cases excluded by the human reader were also excluded by the AI platform.

**Figure 3:**
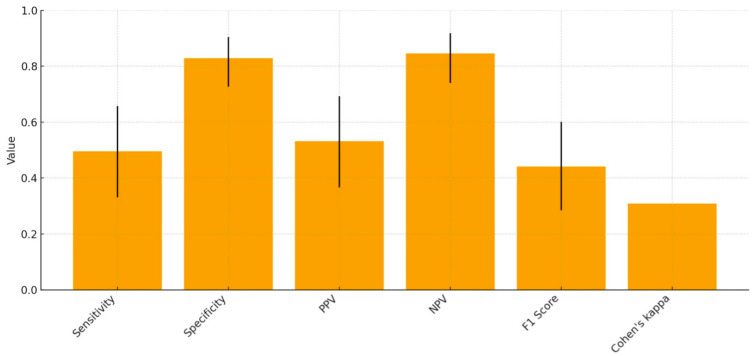
Diagnostic accuracy metrics with 95% confidence intervals for AI-based angle Class assessment.

When the N/A category was included as a valid diagnostic outcome, the overall diagnostic accuracy—defined as the proportion of identical classifications between AI and reader—was 47.5% (28/59 cases). A summary of the diagnostic accuracy metrics used in the dental class assessment is presented in Table 3 and Figure 3.

### 3.7. Intra-Observer Reliability

The intra-observer reliability for the reference cephalometric measurements was excellent, with an ICC range of 0.92–0.98 for angular measurements and a Kappa of 0.90 for categorical classifications.

## 4. Discussion

In this study, we evaluated the diagnostic reliability of the Diagnocat platform for orthodontic assessment via CBCT, comparing its categorical outputs with those of a reference 2D cephalometric analysis. The findings demonstrate that the current version of the software provides limited diagnostic information for skeletal and vertical pattern evaluation and exhibits only fair agreement with the human examiner in terms of Dental Angle classification. These results suggest that, in its present form, the platform is not sufficiently reliable to support independent orthodontic diagnosis based on CBCT data alone.

The most significant finding of this study was the AI’s failure to generate a diagnosis in >90% of skeletal and vertical assessments. This phenomenon likely reflects the algorithm’s internal “confidence threshold.” While this approach minimizes false positives (high specificity), it drastically reduces sensitivity and utility. In this study, the AI behaved with extreme conservatism. It appears the system is calibrated to withhold judgment on any anatomy that deviates even slightly from its training “ideal,” rendering it ineffective for orthodontic patients who, by definition, possess deviant anatomy.

The most notable limitation of the assessed AI tool was its inability to generate categorical skeletal or vertical diagnoses in most cases (>90%). While all three cases with available AI skeletal outputs were concordant with the reference analysis, the extremely low rate of analyzable predictions prevented meaningful assessment of diagnostic accuracy. Similarly, only one hyperdivergent case was identified by the AI in the vertical analysis, despite nearly 42% of patients being classified as hyperdivergent by the reference method.

Clinically, the “N/A” outputs cannot be interpreted as neutral results; they likely reflect internal confidence thresholds, technical processing limitations, or anatomical patterns outside the algorithm’s training distribution. Regardless of the internal cause, from a clinician’s perspective, an “N/A” output represents a functional system failure, as it does not support diagnosis, decision-making, or workflow efficiency. In real-world clinical settings, such outputs increase cognitive and workflow burden by requiring manual verification and repeat analysis, thereby negating the intended automation benefit of AI-assisted diagnostics.

By contrast, Dental Angle classification was provided in more than half of the cases and showed fair agreement (κ = 0.31) with the reference examiner. The algorithm demonstrated relatively high specificity and NPV, suggesting a conservative decision threshold designed to avoid overdiagnosis; however, this conservative approach also resulted in a substantial number of unclassified cases.

There was high agreement with the reference method (100% in the few evaluable skeletal and vertical cases) when the AI provided a diagnosis, but the proportion of analyzable studies was unacceptably low. These findings align with prior reports emphasizing that many commercial AI tools in orthodontics remain “black boxes,” offering limited transparency in terms of algorithmic logic and confidence estimation [17,18].

The lack of transparency inherent to proprietary “black-box” AI systems raises important ethical and practical concerns for clinical adoption. When diagnostic outputs cannot be interrogated, audited, or explained, responsibility for errors remains entirely with the clinician, despite algorithmic involvement in the diagnostic process. This asymmetry between algorithmic authority and human accountability creates ethical tension, particularly when systems provide incomplete outputs without explainability. Trust in clinical AI systems depends not only on accuracy but also on transparency, interpretability, and predictable behavior; features that are currently absent in many commercial platforms.

Among all the diagnostic tasks, the Dental Angle class evaluation achieved the most consistent results. When all valid cases (classes I–III and N/A) were considered, the AI reached an overall diagnostic accuracy of 47.5%, with Cohen’s κ = 0.31, indicating fair agreement with the reference reader. The system demonstrated relatively high specificity (0.83) and NPV (0.85), suggesting conservative decision thresholds but only moderate sensitivity (0.50). This pattern suggests that the algorithm tends to avoid false positives by withholding uncertain classifications, resulting in an excess of “nonclassifiable” outcomes. Such conservative bias is typical for commercial AI models that prioritize safety over coverage in clinical diagnostics and has already been reported in a few validation studies [19,20]. This behavior of the program may be attributable to its internal configuration, which prioritizes minimizing false-positive errors but, as a consequence, yields a substantial number of missed or no diagnoses.

Despite our findings, several studies have shown that AI frameworks, particularly those utilizing convolutional neural networks (CNNs), can increase the accuracy and efficiency of diagnosing malocclusions and detecting specific craniofacial pathologies [21,22]. A narrative review by Azizi et al. [22] highlights multiple studies where CNNs have been effectively employed to classify malocclusions from dental images, achieving accuracy rates exceeding 93% in assessments of skeletal malocclusions [23,24]. This capability indicates that AI not only augments the diagnostic process for orthodontists but can also lead to more precise treatment planning.

Our findings contrast with recent reviews by Azizi et al. [22] and others who report >93% accuracy for CNNs. However, a critical distinction must be made between research-grade AI systems and commercial AI platforms. Research models are typically trained and validated on curated, balanced datasets with controlled imaging protocols and optimized annotations, often under idealized conditions. By contrast, commercial systems operate on heterogenous real-world clinical data, across varying acquisition protocols, anatomical variability, and pathology distributions. Therefore, performance metrics reported in experimental AI studies cannot be directly extrapolated to commercial clinical deployment. Our findings highlight this translational gap between algorithmic performance in controlled research environments and functional reliability in routine clinical workflows.

Previous research on AI-driven orthodontic software has focused primarily on landmark-based cephalometric analyses rather than categorical diagnoses. Studies involving commercially available platforms such as AudaxCeph, CephX, and WebCeph have shown promising results, with high agreement (ICC > 0.90) between AI and human measurements of 2D data [5]. However, 3D-based orthodontic analysis remains more challenging. Bao et al. [25] performed AI-automated cephalometric analysis of CBCT-derived reconstructed lateral cephalograms, which showed that automated analysis was not yet accurate enough to replace human analysis. Serafin et al. [26] reported an average discrepancy of 2.44 mm between automated and manual localization of cephalometric landmarks in three-dimensional datasets. Their meta-regression revealed a significant association between publication year and the magnitude of tracing error, suggesting a temporal trend toward improved performance. The authors attributed these gains to advances in AI systems, concluding that contemporary algorithms achieve high accuracy in landmark detection.

For the clinician, a “No Diagnosis” output is not neutral; it is disruptive. Reliance on an automated system that fails 9 times out of 10 creates a workflow bottleneck, requiring the clinician to verify the failure and then manually perform the analysis they hoped to automate. Therefore, in its current v1.0 state, the tool cannot act as a triage system or a second opinion, as it remains silent on the vast majority of malocclusions. Although the Diagnocat platform demonstrated acceptable specificity in Dental Angle classification, the overall level of diagnostic completeness was insufficient to support clinical decision-making. In routine orthodontic practice, incomplete outputs may lead to workflow delays or the need for manual overriding of AI-generated reports. Nevertheless, the platform’s automated data processing, integration with CBCT datasets, and structured reporting capabilities may offer practical value once its orthodontic diagnostic modules undergo further refinement.

From a clinical standpoint, Diagnocat’s performance in Dental Angle classification and overbite assessment (κ = 0.32) may support preliminary triage or educational applications but is insufficient for diagnostic decision-making. The software’s high proportion of missing classifications could delay rather than streamline the orthodontic workflow. Nonetheless, the platform’s integration with radiological data management systems and automated report generation remains a practical advantage, suggesting potential value once diagnostic reliability improves. This is likely attributable to the need for further refinement of the Diagnocat AI platform, which is currently capable of generating automated reports for both three-dimensional cone-beam computed tomography (CBCT) scans and two-dimensional panoramic and intraoral radiographs. Despite numerous positive findings regarding Diagnocat’s diagnostic reliability in other domains [27,28,29], the introduction of an orthodontic module at its present stage of development appears premature.

Recent evidence underscores that AI-based 3D cephalometry can achieve close agreement with manual tracing under defined conditions, but performance is tool- and workflow-specific. For example, Khabadze et al. compared AI-assisted 3D cephalometry with manual tracing and reported statistically significant differences for selected parameters, emphasizing that agreement may vary by measurement and implementation [30]. In parallel, an umbrella review of systematic reviews concluded that, despite progress, automatic cephalometric landmark identification has not yet reached a consensus for unsupervised clinical use across settings [31]. These findings align with our decision to focus on independent validation of a commercial system’s clinically presented outputs and completeness rather than assuming upstream landmark accuracy.

The strengths of this study include a clearly defined diagnostic accuracy framework, blinding between AI and reference assessments, and the use of real-world CBCT examinations from an orthodontic population. The study design reflects the actual clinical workflow, in which the Diagnocat platform is intended by its developers to serve as an integral component.

However, the study has certain limitations. First, the sample size was relatively small, which may have limited the robustness of certain subgroup analyses. Another limitation is the reliance on the evaluation of a single investigator; although this approach may approximate real-world clinical workflows, it can still introduce bias. The AI system’s internal algorithms, training data, and confidence thresholds were undisclosed by its producer, preventing technical analysis of failure modes. Furthermore, we evaluated only one AI version (Diagnocat 1.0), and subsequent updates may have altered performance. Finally, the high proportion of AI “no-diagnosis” outcomes restricted agreement metrics from being assessed for several diagnostic categories.

Future studies should explore multicenter datasets with larger and more diverse populations, evaluate multiple AI versions over time, and analyze the impact of different CBCT acquisition protocols on algorithm performance. Additionally, transparent reporting of confidence levels or heatmaps may help clinicians better understand the basis of AI-generated predictions and improve trust and usability. Comparative studies between multiple commercial tools may further clarify the current landscape of AI-based orthodontic diagnostics and inform the development of clinically robust algorithms.

From a clinical perspective, while the platform is not suitable for independent diagnostic decision-making in its current form, it may still offer value under restricted conditions. Potential applications include educational use, preliminary screening, structured reporting assistance, or supervised clinical workflows where AI outputs are used as supportive information rather than a diagnostic authority.

With improvements in data completeness, transparency, and confidence calibration, such platforms could evolve into reliable triage or decision support systems. At present, however, human oversight remains essential for all orthodontic diagnostic interpretations generated by commercial AI systems.

## 5. Conclusions

In this independent validation study of Diagnocat v1.0, the orthodontic module showed inadequate clinical usability for CBCT-based categorical orthodontic reporting. This is primarily due to its extremely low completeness for skeletal and vertical outputs and only fair agreement for overbite and Angle class classifications. These findings apply to the tested commercial system/version and output definitions; they should not be generalized to CBCT-based orthodontic diagnosis or AI cephalometry as a whole. Transparent reporting of computational basis and confidence metrics, together with larger multicenter, multi-reader validations, is required before such tools can be relied upon for clinical decision-making.

## Figures and Tables

**Table 1:** Contingency table for sagittal skeletal classification.

	Reference Class I	Reference Class II	Reference Class III	Total (AI)
AI Class I	1	0	0	1
AI Class II	0	1	0	1
AI Class III	0	0	1	1
AI (no diagnosis)	20	28	8	56
Total (Reference)	21	29	9	59

**Table 2:** Contingency table for overbite classification.

	Reference Decreased (<2 mm)	Reference Normal (2–4 mm)	Reference Increased (>4 mm)	Total (AI)
AI Decreased (<2 mm)	11	14	10	35
AI Normal (2–4 mm)	1	0	0	1
AI Increased (>4 mm)	1	4	5	10
AI (no diagnosis)	2	8	3	13
Total (Reference)	15	25	19	59

**Table 3:** Diagnostic accuracy metrics of the AI system for angle Class assessment (including N/A).

	Sensitivity	Specificity	PPV	NPV	F1 Score	Cohen’s κ
Value	0.475 (0.331–0.658)	0.828 (0.727–0.905)	0.532 (0.366–0.693)	0.846 (0.740–0.918)	0.441 (0.285–0.601)	0.309

PPV—positive predictive value; NPV—negative predictive value; F1—harmonic mean of sensitivity and PPV. 95% CI in parentheses.

## Data Availability

The raw data supporting the conclusions of this article contain patient diagnostic information and are therefore subject to privacy and legal restrictions. The data are available from the authors upon reasonable request.

## List of Abbreviations

2D	two-dimensional
3D	three-dimensional
AI	artificial intelligence
ANB	A–N–B angle (point A–nasion–point B); sagittal jaw relationship metric
AP	anteroposterior
CA	cephalometric analysis
CBCT	cone-beam computed tomography
CI	confidence interval
CNN	convolutional neural network
CT	computed tomography
DAP	dose–area product
DICOM	digital imaging and communications in medicine
F1	F1 score (defined here as the harmonic mean of sensitivity and PPV)
FMA	Frankfort–mandibular plane angle
FOV	field of view
ICC	intraclass correlation coefficient
IQR	interquartile range
κ	Cohen’s kappa (kappa coefficient)
kVp	peak kilovoltage
L1MP	lower incisor to mandibular plane angle
mA	milliampere
mAs	milliampere-seconds
N/A	not applicable/no diagnosis (noninterpretable output)
NAPog	N–A–Pog angle (facial convexity)
NPV	negative predictive value
PPV	positive predictive value
SaaS	software as a service
SD	standard deviation
SE	standard error
SNA	S–N–A angle (sella–nasion–point A); maxillary sagittal position metric
SNB	S–N–B angle (sella–nasion–point B); mandibular sagittal position metric
SNGoGn	sella–nasion to gonion–gnathion angle (vertical skeletal pattern metric)
SNMP	sella–nasion to mandibular plane angle
STARD	standards for reporting diagnostic accuracy studies
U1SN	upper incisor to sella–nasion angle
Wits	Wits appraisal (AO–BO; sagittal jaw disharmony metric)
Y-axis	Y-axis angle (growth axis/vertical pattern descriptor)
